# The WISDOM Study: breaking the deadlock in the breast cancer screening debate

**DOI:** 10.1038/s41523-017-0035-5

**Published:** 2017-09-13

**Authors:** Laura J. Esserman, the WISDOM Study and Athena Investigators, Hoda Anton-Culver, Alexander Borowsky, Susie Brain, Thomas Cink, Beth Crawford, Martin Eklund, Laura Esserman, Joshua Fenton, Diane Heditsian, Robert A. Hiatt, Michael Hogarth, Celia Kaplan, Barbara Koenig, Andrea LaCroix, Kathryn M. Larsen, Vivian Lee, Jeffrey Matthews, Lisa Madlensky, Arash Naeim, Haydee Ojeda-Fournier, Barbara A. Parker, Karen Sepucha, Yiwey Shieh, Allison Stover Fiscalini, Carlie Thompson, Jeffrey Tice, Laura Van ‘T Veer, Neil Wenger, Elad Ziv

**Affiliations:** 2 Department of Epidemiology, University of California, Irvine, CA USA; 3 Center for Comparative Medicine, University of California, Davis, CA USA; 4 Breast Oncology Program, University of California, San Francisco, CA USA; 5 Department of Radiology, Sanford Health, Iowa, IA USA; 6 Division of Genomic Medicine, University of California, San Francisco, CA USA; 7 Department of Medical Epidemiology and Biostatistics, Karolinska Institute, Solna, Sweden; 8 Department of Surgery & Radiology, University of California, San Francisco, CA USA; 9 Family & Community Medicine, University of California, Davis, CA USA; 10 Breast Oncology Program, University of California, San Francisco, CA USA; 11 Department of Epidemiology and Biostatistics, University of California, San Francisco, CA USA; 12 Department of Pathology and Laboratory Medicine, University of California, Davis, CA USA; 13 Medical Effectiveness Research Center, University of California, San Francisco, CA USA; 14 School of Nursing, University of California, San Francisco, CA USA; 15 Division of Epidemiology, University of California, San Diego, CA USA; 16 Department of Family Medicine, University of California, Irvine, CA USA; 17 Breast Oncology Program, University of California, San Francisco, CA USA; 18 Scientific Consultants, North Vancouver, BC Canada; 19 Department of Family Medicine and Public Health, University of California , San Diego, CA USA; 20 Biomedical Informatics, University of California, Los Angeles, CA USA; 21 Department of Radiology, University of California, San Diego, CA USA; 22 Department of Hemotology/Oncology, University of California, San Diego, CA USA; 23 Health Decision Sciences Center, Massachussetts General Hospital, Boston, MA USA; 24 General Internal Medicine, University of California, San Francisco, CA USA; 25 Department of Surgery, University of California, San Francisco, CA USA; 26 Department of Surgery, University of California, San Francisco, CA USA; 27 General Internal Medicine, University of California, San Francisco, CA USA; 28 Breast Oncology Program, University of California, San Francisco, CA USA; 29 General Internal Medicine, University of California, Los Angeles, CA USA; 30 Institute for Human Genetics, University of California, San Francisco, CA USA; 1 Department of Surgery and Radiology, University of California, San Francisco, CA USA

## Abstract

There are few medical issues that have generated as much controversy as screening for breast cancer. In science, controversy often stimulates innovation; however, the intensely divisive debate over mammographic screening has had the opposite effect and has stifled progress. The same two questions—whether it is better to screen annually or bi-annually, and whether women are best served by beginning screening at 40 or some later age—have been debated for 20 years, based on data generated three to four decades ago. The controversy has continued largely because our current approach to screening assumes all women have the same risk for the same type of breast cancer. In fact, we now know that cancers vary tremendously in terms of timing of onset, rate of growth, and probability of metastasis. In an era of personalized medicine, we have the opportunity to investigate tailored screening based on a woman’s specific risk for a specific tumor type, generating new data that can inform best practices rather than to continue the rancorous debate. It is time to move from debate to wisdom by asking new questions and generating new knowledge. The WISDOM Study (Women Informed to Screen Depending On Measures of risk) is a pragmatic, adaptive, randomized clinical trial comparing a comprehensive risk-based, or personalized approach to traditional annual breast cancer screening. The multicenter trial will enroll 100,000 women, powered for a primary endpoint of non-inferiority with respect to the number of late stage cancers detected. The trial will determine whether screening based on personalized risk is as safe, less morbid, preferred by women, will facilitate prevention for those most likely to benefit, and adapt as we learn who is at risk for what kind of cancer. Funded by the Patient Centered Outcomes Research Institute, WISDOM is the product of a multi-year stakeholder engagement process that has brought together consumers, advocates, primary care physicians, specialists, policy makers, technology companies and payers to help break the deadlock in this debate and advance towards a new, dynamic approach to breast cancer screening.

## Introduction

Annual screening mammography—the most common approach in the US today—has its roots in the large, randomized screening trials of the 1980s.^1^ The first trial of annual screening, the U.S. Health Insurance Plan of Greater New York, began in 1963 and included 31,000 women in each arm.^2^ At 18 years of follow-up, it showed a 25% reduction in mortality, although benefit to women in their forties accrued after they were 50. The overview of the Swedish trials of bi- or triennial screening showed a relative reduction in breast cancer mortality of 21%, with maximum benefit for women in their sixties.^3^ The degree and timing of benefit to younger women in particular has generated a great deal of controversy.^4^ Even a decade later, there remains a continuing debate over the methodologic flaws of each of these studies, the net effect of which has impeded consensus on public recommendations for breast screening.^5–7^

From the outset, translating these studies into population-based screening recommendations stirred controversy, with debate focused on the frequency and most appropriate age to begin screening. The January 1997 Consensus Development Panel convened by the National Institutes of Health recommended women aged 40–49 be informed of the benefits and risks of screening and decide for themselves.^8^ The National Cancer Institute (NCI) and American Cancer Society (ACS), however, recommended regular screening for women in their forties while disagreeing on screening frequency, with the former recommending every 1–2 years, the latter annually. Partly because of the controversy generated, the NCI later stopped issuing screening guidelines.

Now, 20 years later, we find ourselves in a familiar place—still reviewing and reanalyzing data from the same trials, debating the optimum age to begin and interval, with professional societies that set guidelines compelled to “take a side” in the debate. The controversy following the 2009 JAMA commentary “Rethinking Screening”^9^ and updates to USPSTF guidelines^10^ illustrates how entrenched both sides have become. Consensus on recommendations remains distant.

The US Preventative Task Force systematic review concluded in 2015,^11^ much like it had in 2009,^10^ that mammographic screening benefits women over 50 and that biennial, not annual, screening was recommended for women ages 50–74. After weighing the balance of harms and benefits for women aged 40–49, screening was not recommended routinely for women in their forties. Instead, the USPSTF suggested an individualized approach taking patient risk and personal preference into account. In contrast, 2017 guidelines from the American College of Radiology and the Society of Breast Imaging currently recommend annual screening starting at age 40.^12^ The American Cancer Society has revised their guidelines and recommend annual mammograms for women over 45 of average risk, with women between the ages of 40–44 provided the opportunity to begin annual screening. Women over the age of 55 are recommended to receive biennial screening, although annual screening may be considered.^13^

Although the academic debate has progressed little in these 30 years, what has changed is public awareness of this issue. The vast press coverage of the Canadian National Breast Cancer Screening Study (CNBCSS) in 2014 brought the potential harms of screening into the public spotlight. Not only did the 25-year analysis of the CNBCSS show, as it had at both 10 and 15 years, that annual screening failed to reduce breast cancer mortality, it provided the first-ever estimates of overdiagnosis in a population-based annual screening program: half of all screen-detected non-palpable cancers were estimated to be indolent lesions that would otherwise never have come to clinical attention.^14^

For some, this result simply confirmed previous findings showing that up to 20% of all cancers (50% of screen-detected cancers) fall into the category of overdiagnosis^15–17^—meaning a woman has a greater chance of being over-diagnosed than of having her life saved by screening.^18^ Others pointed to studies demonstrating there is little, if any overdiagnosis in breast cancer,^19, 20^ labeling CNBCSS as a flawed analysis or simply the latest way to attack screening. The debate reached such an unhealthy tenor that published exchanges even included accusations of a scientific conspiracy to reduce access to mammography.^21, 22^

Like the screening debates that preceded it, the controversy surrounding overdiagnosis has now settled into a familiar pattern. It focuses on largely technical arguments over statistical assumptions, corrections for lead time bias and varied demographics^23, 24^ that create uncertainties in the data and ultimately have shifted the debate into the realm of opinion, rather than fact. Recent characterization of a molecular profile to define an ultralow risk biology may provide a tool to more objectively categorize ultralow risk breast cancers that have little systemic risk of progression. This is an important advance that may help us to improve our ability to treat the disease and further tailor individual screening recommendations.^25^

We must remember, however, that the data we are arguing over are from decades-old trials, from an era before most of the effective systemic therapies were available. That there is an impact of modern systemic therapies on reducing breast cancer mortality is undeniable—some estimate that systemic therapy accounts for 2/3 of the observed reduction in mortality.^26, 27^ The rise of endocrine therapy^28, 29^ may also mitigate the impact of finding some cancers later.

Whether one believes these figures or not, the takeaway is that we are stuck in an endless cycle of academic debate, arguing over data that have little context in the modern treatment setting. Breast cancer treatment continues to rapidly evolve towards a patient-centered, precision medicine approach that recognizes what is perhaps the most important lesson we have learned over the past two decades of research: that breast cancer is not a monolithic entity, but a spectrum of disease. From indolent lesions of epithelial origin (IDLE)^9^ requiring no treatment, to aggressive disease requiring equally aggressive treatment, it has resisted all our attempts to lump it into a single bin.

Yet we continue our one-size-fits-all approach to breast cancer screening. It is contrary to the very nature of the disease. We cannot continue to focus the entirety of our efforts on a screening approach that is based on an outdated understanding of breast cancer biology, expecting that the uncertainties and debate will finally be resolved. Instead, we must be willing to innovate and to entertain new paradigms of screening that incorporate our current understanding of breast cancer, its treatment and risk susceptibility by putting them to the test.

We may have little choice. Because the consequences of failing to do so may be to further alienate the very women screening is supposed to help.

## What women want: better, not more screening

Even though generations of women educated in the benefits of screening mammography generally regard it positively, experience shows it is a fragile trust. A single false positive can cause psychological distress for up to 3 years and reduce adherence to subsequent screening by 37%.^30–34^

Considering the specificity of mammography is generally accepted to be ~ 90% (e.g., 1 in 10 are false positive), whereas the real breast cancer rate is ~ 5 in 1000 women, the majority of abnormal mammograms are, in fact, false positives. After 10 years of annual screening, over half of all women receive a false-positive recall and 7–9% have a false-positive biopsy.^35^

Furthermore, in the wake of the CNBCSS, information concerning overdiagnosis is increasingly available to women,^36^ undermining their confidence in screening. Women given controlled, qualitative, and quantitative education on the risks of overdiagnosis have less positive attitudes about screening and demonstrate reduced intent to screen.^37^ Similarly, primary care physicians, key influencers in a woman’s screening decisions, are far less willing to refer patients 40–49 for screening when fully educated about the potential risks/benefits of screening.^38^

Further, our conflicting recommendations have made this divisive debate a public one, sowing distrust and a deepening confusion for women over how to prevent the disease that scares them the most.^39, 40^

The question we need to be asking, therefore, is not whether we should screen more or less, earlier or later. It is how can we make screening better for women, reduce false-positive recalls and improve our ability to more accurately prevent and detect clinically significant cancers sufficiently early. This is, after all, is what women tell us they want^41^ and what we have observed to date in the WISDOM trial (described below).

The answer is simply that we must move on. We must begin developing and testing new and better approaches that respond to women’s needs. Fortunately, in this respect, there is one thing upon which we all agree—women must have the opportunity to make informed screening choices.

## Individualized, informed choice

Overwhelmingly, women want information about their personal risk of breast cancer.^42, 43^ Currently, only 10% have accurate perceptions of their personal risk and 40% have never discussed their personal breast cancer risk with a doctor.^44^ Yet, a realistic view of their risk is prerequisite to making informed screening decisions.

We have the tools to better inform women of their personal risk, through well characterized models that incorporate family history and breast density, endocrine exposures, gene mutations, and atypia,^45–49^ along with a number of common gene variants.^50, 51^ They teach us that not all women whom we classify for screening purposes as “average-risk,” actually have the same lifetime risk of breast cancer. Armed with a better understanding of their individual risk, such women will expect—and demand—screening recommendations commensurate with their personal risk.

Unless we are prepared to ignore the modern tools available to us, we are therefore compelled to shepherd breast cancer screening into the era of precision medicine. Now is the time to begin evaluating a patient-centric model, focusing on individually tailored recommendations on when to start, when to stop, and how often to screen, depending upon a woman’s personal risk. Only through clinical testing can we establish the evidence that tells us how best to apply risk.

The idea of risk-based screening is not revolutionary. In fact, we already do it, although in a crude fashion. It is standard of practice for high risk women with mutations in the BRCA genes and first degree relatives from high risk families to begin screening at a much earlier age, and to do so more frequently (annual mammogram alternating with annual MRI).^52^ But our understanding of breast cancer risk goes much further than our current screening recommendations reflect. Our failure to incorporate our current understanding of personal risk into our screening recommendations means we may be asking some women to accept risk/benefit ratios they might not be comfortable with if they were fully informed.

Within the context of well-designed, randomized, controlled clinical trials, we have the ability to investigate new screening models in a safe, systematic manner, beginning with conservative estimates that minimize the chances of misclassification of risk and avoid underscreening. If we are successful, it could help establish a new baseline for cancer screening, reduce confusion and anxiety for women over conflicting recommendations, improve women’s perception of their true risk of breast cancer, improve adherence,^53^ and reinforce confidence in providers. Perhaps most importantly, it allows us to learn who is at risk for what kind of cancer, and establish a cycle of continuous improvement in breast cancer screening.

Risk-based screening may or may not be the answer to all of screening’s shortcomings, but it is perhaps an answer to the current deadlock in which we find ourselves. In the words of philosopher David Hume, it is time “we start spilling our sweat, and not our blood.”

## The WISDOM study: overcoming challenges

**Fig. 1:**
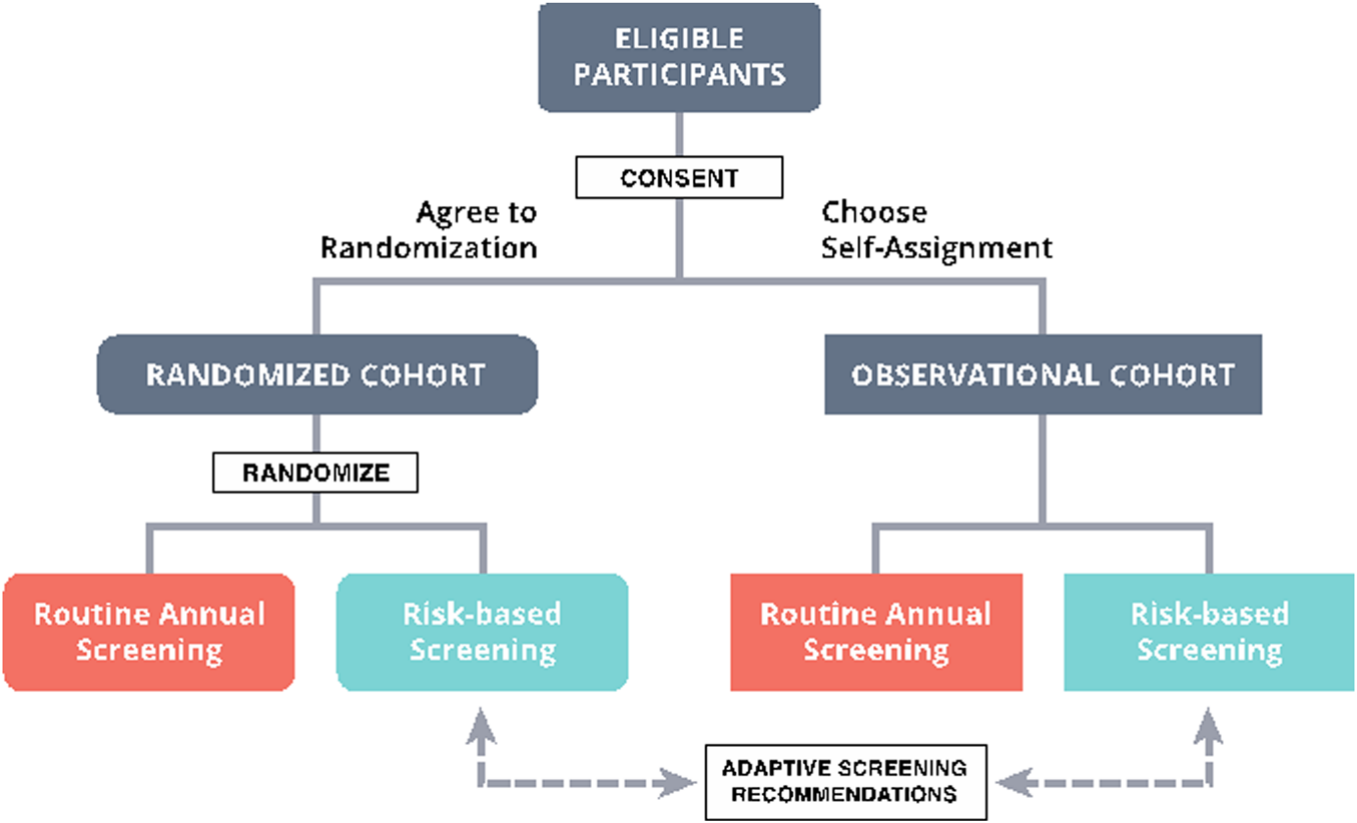
Overall WISDOM study schema

We have recently been awarded a grant from the Patient Centered Outcomes Research Institute (PCORI) to evaluate a risk-based screening approach within a pragmatic, controlled trial. The “WIDSOM” study (Women Informed to Screen Depending On Measures of risk) is a multicenter trial comparing risk-based screening to annual screening in 100,000 women aged 40–74, initially opening in the Athena Breast Health Network in California and the Midwest (Table 1). The study is a “preference tolerant design” (Fig. 1) that encourages women to be randomized (*n*~ 65,000) but also allows self-assignment for those with strong personal preference for either annual or risk-based screening (a pilot study was conducted in 2015 in which 74% of women agreed to randomization). Importantly, WISDOM is an adaptive design, allowing us to learn and adjust, continuing to improve the risk-assessment and screening recommendation models over the course of the trial.

**Table 1:** The WISDOM Study At-a-glance

Rationale	• In >30 years of screening, little change in approach
	• No clear evidence that annual mammograms reduce breast cancer mortality rates compared to biennial mammograms
	• Morbidity associated with annual screening—false positives, over-diagnosis/overtreatment of indolent disease—could safely be reduced
	• Conflicting screening recommendations for women in their 40’s has resulted in confusion for patients, who want more personalized advice
Hypotheses	Personalized breast cancer screening recommendations based on individual risk assessments will: (1) be at least as safe and less morbid than annual screening; (2) result in improved breast cancer prevention; and (3) be readily accepted by women and preferred over standard annual screening.
Primary endpoint(s)	(i) Safety: comparative rate of stage IIB cancers or higher diagnosed in annual vs. risk-based screening arms (non-inferiority); and
	(ii) Morbidity: reduced rate of recall and breast biopsy between arms
Secondary endpoint(s)	• Rate of stage IIB and interval cancers
	• Recall rates and follow-up procedures
	• Rates of DCIS
	• Rates of chemoprevention use and cancer incidence
	• Proportion of women enrolling in randomized cohort vs. self-assigned cohort
	• Within the self-assigned cohort, proportion of women choosing risk-based screening vs. standard annual screening to assess preference
	• Adherence to assigned screening schedule
	• PROMIS anxiety score
	• Breast cancer risk worry
	• Decision regret
	• Rates of systemic therapy (comparative differences in treatment stratified by tumor type)
	• Distribution of biological tumor subtypes, including ultralow risk and DCIS
Integral biomarkers:	Components of individual risk assessment^a^:
	(i) Breast Cancer Surveillance Consortium (BCSC) 5-year risk which includes:
	• Age
	• Race/ethnicity
	• First degree relatives with breast cancer
	• Prior breast biopsies (+ve or –ve)
	• Proliferative breast condition (atypia)
	• BI-RADS breast density score
	(ii) Genomic tests for rare high/moderate-penetrance mutations in a number of genes, including the following: BRCA1, BRCA2, ATM, CDH1, CHEK2, PALB2, PTEN, STK11 and TP53
	(iii) Polygenic risk score from 96 lower-risk common genetic variants (SNPs) with known association to breast cancer (updated as data as data emerges)
	(iv) 10 year life expectancy as assessed by e-prognosis
	(v) Special risk factors such as history of chest irradiation
Integrated biomarkers^a^:	(i) Updated polygenic risk model (including ethnicity and race specific SNPs that are shown to confer risk (258 under assessment)
	(ii) Risk factors associated with subtype specific breast cancer, including ultralow risk or indolent lesions, molecular subtypes, interval cancers
Sample size (and power)	Sample size:
	Total of 100,000 participants (~ 65,000 in randomized cohort, ~ 35,000 in self-assigned cohort)
	Statistical power:
	• 90% to detect a <0.05% difference in risk of diagnosis with Stage IIB or higher in the personalized vs. annual arm in a given year
	• 90% power to detect a difference in number of recall biopsies as small as 1.1% between personalized vs. annual arm
Patient population (3 bullets)	• Women, aged 40–74 years at enrollment
	• No prior history of cancer or DCIS
	• Willing to sign informed consent and provide follow-up data

^a^ As risk models improve over time, the optimal risk model will be updated and used for risk assignments, as we are testing the concept of risk-based screening, not simply a specific risk model

An essential aspect of developing WISDOM has been the engagement of all stakeholders, including consumers, policy makers/guideline organizations and multiple specialties, and payers, to agree upfront on metrics for success. This ensures the trial remains relevant to the needs of the end-user and sets the stage for rapid adoption should it prove successful.

Patients and advocates in particular, through the Athena Consumer and Community Advisory Committee, have been key partners in WISDOM since its conception. The preference-tolerant design that allows all women to participate regardless if they have strong personal reservations about being randomized grew from vigorous discussions with this group. The consumer voice is deeply embedded in WISDOM, with influence in all aspects of study design and planning, including enrollment strategies, consent processes, primary care physician outreach and education, risk notification, and participant retention.

The buy-in of health care payers is essential to enable rapid dissemination once results are presented. Modeling shows a risk-based strategy will be more cost effective in terms of screening, but requires an initial outlay of resources for one-time genetic testing and comprehensive risk assessments. After almost 2 years of discussion and negotiation, WISDOM’s “Payer Working Group”, led by Blue Shield of California and including all insurers in California, has reached an agreement to implement a “Coverage with Evidence Development” model to cover clinical costs not funded through PCORI.^54^ This model allows innovative treatment approaches to be tested transparently. The use of a coverage model that fosters the development of evidence, using a coverage with trial participation approach, allows agreement on metrics for adoption and should shorten the timeline for adoption. By engaging payers early, should the study prove successful, we will have laid the foundations to address future challenges in implementation related to standard coverage. We are in the process of engaging other payers.

The most formidable challenge in terms of stakeholders in developing a trial of risk-based screening, given the voracity of the academic debate, lies within the academic community. Among the highest priorities has been to define acceptable parameters of risk assessment, stratification and screening recommendations. We have also invested considerable effort to reach consensus regarding what constitutes success. WISDOM’s ‘Risk Thresholds Group’ and ‘Primary Care Physician Working Group,” consisting of primary care teams, representatives of the radiology community and others have shared these tasks.

## Risk assessments and recommendations

The Breast Cancer Surveillance Consortium (BCSC) model was selected as the foundation of individual risk assessments for WISDOM, based on its accuracy, ease of implementation, its large (>1 million women) multiethnic target population, and incorporation of ethnicity and breast density as risk factors.^55, 56^ Additional assessments also include polygenic risk based on nearly 200 SNPs, as well as a 9 high-penetrance gene mutation panel.

In translating individual risk to screening recommendations, the primary consideration of the working groups was to develop guidelines that were sufficiently conservative to minimize risk of potential harm from underscreening, yet progressive enough to minimize potential harm from overdiagnosis, while permitting outcome measures with sufficient study power. The consensus risk stratification and related screening recommendations to be employed within WISDOM are shown in Table 2, and include more frequent screening for those at highest risk or those at risk for faster growing (e.g., hormone negative) cancer. In the risk-based assessment arm, no woman will receive a recommendation for less screening than current USPSTF guidelines—individual risk ≥ 1.3% over 5 years initiates screening. Because the uptake of risk-reducing interventions has been very poor despite level 1 evidence of benefit, we will use a stringent threshold (top 2.5 percentile of risk for breast cancer by age group, or lifetime risk in the range of 30% or higher) for identifying participants to target and counsel about endocrine risk-reducing therapy. The gene-based tests also inform the risk for hormone positive or negative breast cancer and impact screening and prevention recommendations. Additional details on the rationale and evidence used to develop this model are published elsewhere.^57, 58^

**Table 2:** WISDOM US risk stratification and screening recommendations

Risk	Highest risk	Elevated risk	Average risk	Lowest risk
Criteria/threshold	BRCA1/2, TP53, PTEN, STK11, CDH1 mutation carrier	Women aged 40–49 with extremely dense breasts	Women aged 50–74	Women aged 40–49 with a <1.3% 5-year risk of developing breast cancer
	or	or	or	
	ATM, PALB2, or CHEK2 mutation carrier with positive family history of breast cancer	Women at a ≥ 0.75% 5-year risk of developing ER-breast cancer based on susceptibility, age, and ethnicity	Women aged 40–49 with a ≥ 1.3% 5-year risk (risk of an average 50 year-old woman)	
	or	or		
	Women with a ≥ 6% 5-year risk (risk of an average BRCA carrier)	Women in top 2.5th percentile of risk by 1-year age category		
	or	or		
	Women with a history of mantle radiation between ages 10–30 years	ATM, PALB2 or CHEK2 mutation carrier without a positive family history^a^ of breast cancer		
Screening rec:	Annual mammogram + MRI	Annual mammogram^b^	Biennial mammogram^c^	No screening until age 50

^a^ Family history is defined as a first degree relative with breast cancer, two second-degree relatives with breast cancer, or one second-degree relative diagnosed prior to age 45

^b^ If individual does not meet criteria for annual mammogram + MRI

^c^ If individual does not meet criteria for annual mammogram or annual mammogram + MRI

A shared decision-making (e-prognosis) tool based on recent modeling of comorbidity and impact of screening^59^ will be used to identify women unlikely to benefit from screening due to limited life expectancy. These rules will inform our risk assignments, age to start, age to stop, frequency, and appropriate modality of screening. The trial is designed to adapt over time, and refine categorization and screening frequency based on the actual cancer rate and biology of tumors that develop.

## Defining success

If, after completing WISDOM, we are to avoid simply adding additional fuel to the fire of the screening debate, the scientific questions we ask must be well defined and the answers definitive. This is particularly challenging given the nature of the current debate and is further complicated by statistical requirements, population size limitations and the 5-year follow-up limitation of the funding. Such deliberations within WISDOM working groups strengthened the study significantly, emphasized safety as the overriding priority and established a series of outcomes with achievable and highly relevant goals.

WISDOM’s primary endpoints are, first, to determine whether risk-based screening is non-inferior to annual screening for late-stage cancers detected. The outcome is the number of Stage IIB or higher cancers found using personalized vs. annual screening. The study has been powered assuming annual incidence rates of 95 Stage IIB or higher cancers per 100,000 women in each arm.^60^ Over 65,000 randomized patients, this provides 90% power to detect a difference lower than 0.05% in risk of being diagnosed with Stage IIB or higher in the personalized vs. annual arm in a given year (83% for a difference <0.035%).^58^

Second, we will compare the morbidity of personalized vs. annual screening on the basis of the number of biopsies performed. Assuming 16% of first time mammograms and 8% of subsequent screens lead to false positive recalls,^61^ 65,000 patients equally randomized between annual and personalized screening offers 90% power to detect a difference as small as 1.1% (22 vs. 20.9%).

Additional secondary objectives will further our understanding of the impacts of personalized screening and include measures of morbidity (e.g., rates of systemic therapy, rates of DCIS, chemoprevention) and the comparative attitudes and acceptance of each screening modality by women enrolled in the trial (e.g., adherence, measures of anxiety, decision regret). Finally, we will determine whether an understanding of personalized risk, especially the ability to predict hormone positive breast cancer, will provide better motivation for and uptake of endocrine risk reducing therapies and lifestyle changes.

Since opening in September 2016, Over 4000 women have enrolled in WISDOM. About two-thirds have agreed to randomization. The other one-third opted to self-select their screening approach in the observation arm: 85% have elected for personalized screening. Although preliminary, our experience to date provides critical insight into the comfort women feel with the concept of individualized, risk-based screening.

## Conclusions

The United States is the only country where annual screening starting at age 40 is standard practice, yet our breast cancer mortality rate is no better than countries that screen less.^62^ Clearly, there is room for improvement. Progress will only come by investigating other possibilities. The WISDOM study will evaluate one such possibility—screening based on a woman’s individual risk—opening its first site in August 2016, expanding to other sites nationally in 2017. It is certainly unlikely that all women benefit equally from screening. Investing in pragmatic studies like WISDOM allows us to learn who is at risk for what kind of breast cancer, tailor screening accordingly and build a new framework for continuous improvement.
